# Comparison of early outcomes associated with coronary artery bypass grafting for multi-vessel disease conducted using minimally invasive or conventional off-pump techniques: a propensity-matched study based on SYNTAX score

**DOI:** 10.1186/s13019-022-01905-8

**Published:** 2022-06-07

**Authors:** Lin Liang, Jia-Ji Liu, Qing-Yu Kong, Bin You, Xiao-long Ma, Li-Qun Chi, Jun-ming Zhu

**Affiliations:** Department of Cardiac Surgery, Beijing Anzhen Hospital, Capital Medical University, Beijing Institute of Heart, Lung and Blood Vessel Diseases, No. 2 Anzhen Road, Beijing, 100029 China

**Keywords:** Minimal invasive, Intercoastal space, Multivessel lesion

## Abstract

**Background:**

This study was designed to compare early outcomes associated with coronary artery bypass grafting for multi-vessel disease conducted using either minimally invasive or conventional off-pump techniques.

**Methods:**

From January 2017 through January 2021, 582 patients with multi-vessel lesion coronary artery disease underwent either minimally invasive cardiac surgery coronary artery bypass grafting (MICS CABG) or conventional off-pump coronary artery bypass grafting (OPCABG) treatment by our team at Anzhen Hospital. Patients in the MICS CABG group were propensity score-matched with those in the OPCABG at a 1:1 ratio (MICS CABG = 172; OPCABG = 172), using epidemiological data, preoperative clinical characteristics, and SYNTAX score as covariates. Perioperative outcomes and 6-month computed tomography angiography findings were compared between these groups.

**Results:**

No significant differences between groups were observed with respect to 30-day postoperative mortality, myocardial infarction, and stroke incidence. Surgical data indicated that the MICS CABG procedure was able to cover all three main arterial territories with a relatively low need for circulatory assistance. The MICS CABG procedure was associated with a longer operative duration, but was also associated with higher postoperative hemoglobin and activities of daily living index values as well as a shorter duration of postoperative hospitalization (P < 0.05). No differences in 6-month graft patency were observed between groups.

**Conclusions:**

MICS CABG is a safe, less invasive alternative to OPCABG when performing complete revascularization provided patients are properly selected, yielding similar in-hospital outcomes and 6-month graft patency rates together with an earlier return of physical function.

## Background

While sternotomy-mediated off-pump coronary artery bypass grafting (OPCABG) has been performed for over 30 years and is associated with reductions in morbidity for high-risk patients, conventional sternotomy is nonetheless associated with risks of sternal wound or mediastinal infection, and the postoperative recovery period is generally at least 6–12 weeks long [1–3]. As public demand for minimally invasive surgical procedures continues to grow, a rising number of studies have explored the feasibility of minimally invasive cardiac surgical interventions [4, 5]. Minimal invasive direct coronary artery bypass grafting (MIDCAB) has grown increasingly common as an approach to single left internal thoracic artery (LITA) to left ascending coronary artery (LAD) grafting [6, 7]. In patients harboring multi-vessel lesions or with diffuse coronary artery disease (CAD), some surgeons instead conduct staged hybrid revascularization, others harvest the bilateral internal thoracic arteries for multiple anastomosis [8–11]. As the difficulty of proximal anastomosis has been overcome through 4th or 5th left intercostal incisions, a growing number of studies over the past 20 years have discussed the feasibility of minimally invasive coronary surgery coronary artery bypass grafting (MICS CABG) procedures [12, 13]. MICS CABG surgery has been reported to be less traumatic than conventional OPCABG and is associated with reduced bleeding and more rapid postoperative recovery. Between January 2017 and January 2021, 211 patients underwent multi-vessel MICS CABG at Anzhen Hospital with the goal of achieving complete revascularization via a single left intercostal incision. In this study, we aim to compare the outcomes associated with MICS CABG and conventional OPCABG procedures in a propensity score-matched (PSM) cohort based on SYNTAX score of patients with multi-vessel disease.

## Methods

This was a retrospective single-center study of outcomes associated with multi-vessel grafting. The ethics committee of Beijing Anzhen Hospital approved this study.

### Patient population

From January 2017 to January 2021, 211 patients with coronary angiography results demonstrating multi-vessel disease were treated via MICS CABG by our team at Beijing Anzhen Hospital. During this same period, 371 patients underwent conventional OPCABG via sternotomy at this same center. Patients eligible for inclusion in this study were those with two or more diseased vessels that met the proper indications for OPCABG. Patients were excluded from this study if they: (1) required concomitant valve surgery, ventricle aneurysm repair, or aortic surgery; (2) exhibited severe aortic calcification; (3) suffered from acute myocardial infarction or heart failure; or (4) underwent repeat surgery. All patients signed informed consent forms, and the choice of surgical approach was made with input from both the surgeon and the patient. The ethics committee of Beijing Anzhen Hospital approved this study, and hospital Institutional Review Board approval were obtained to publish these study results.

### Clinical data collection

To evaluate procedure management and clinical characteristics, perioperative clinical details including demographics, clinical outcomes, associated examinations including echocardiographic parameters, chest computed tomography (CT) results, coronary angiography (CAG) results, and complications were extracted from patients medical records. Preoperative data were collected to determine the indications in the MICS CABG and OPCABG groups, and complications in these groups were analyzed. SYNTAX scores can be used as an angiographic grading tool to determine the complexity of coronary artery disease and serve as an invaluable index for patient selection. The pre-discharge Barthel Index is an ordinal scale used to measure performance in 10 activities of daily living (ADL) including feeding, personal toilet use, bathing, dressing and undressing, getting on and off the toilet, bladder control, bowel control, moving from a wheelchair to a bed and back, walking on a level surface (or propelling a wheelchair if unable to walk), and ascending and descending stairs [14].

### Surgical approach

**Fig. 1:**
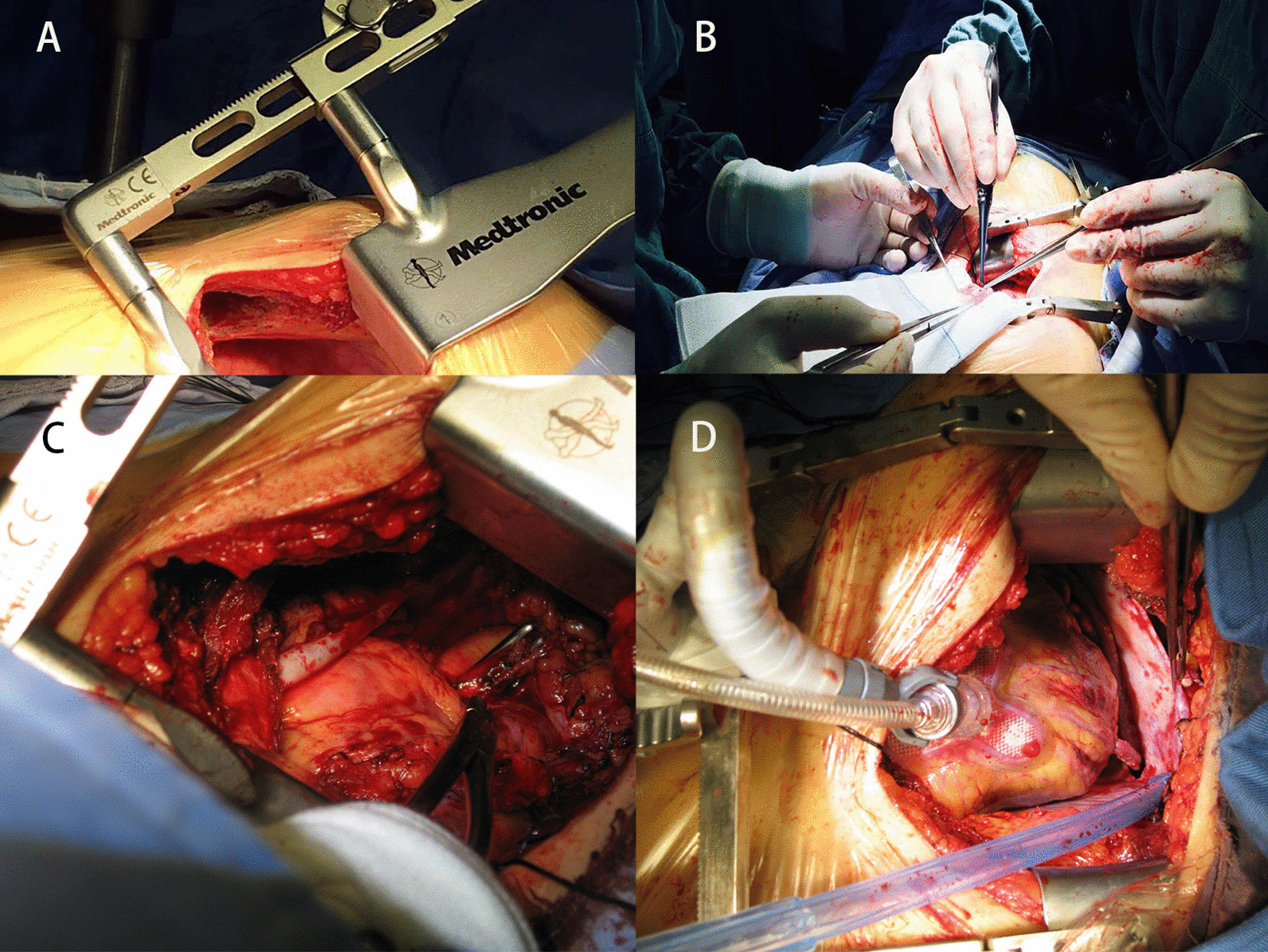
Surgical procedure. **A** LITA retractor (THORATRAK MICS Retractor System) was used to expose the chest wall space for LITA takedown and proximal anastomosis. **B** ValveGate™ Hinged retractor was used for LITA trimming and distal anastomosis. **C** Customized vascular clamp (Cardiomedical GmbH, Langenahgen, Germany) was placed on the ascending aorta for proximal anastomosis. **D** Octopus stabilizer (29800 Medtronic Inc., Minneapolis, MN, USA) was used for the exposure of deeper sites such as the obtuse marginal branch

Conventional OPCABG was performed via sternotomy, and aspirin and clopidogrel were routinely applied. The MICS CABG approach was conducted as follows. Under general anesthesia, selective ventilation of the right lung was achieved with a double-lumen endotracheal tube to achieve LITA exposure for takedown and anastomosis. The left scapula of each patient was padded to elevate it and achieve a rotation of approximately 30° to the right side. A great saphenous vein graft was harvested from the lower extremity, and a left sub-mammary incision (8–12 cm long) provided access to the heart through either the 4th or 5th intercostal space. For female patients, a slightly larger incision was made along the lower edge of the breast tissue. A LITA retractor (THORATRAK MICS Retractor System) was used to expose the chest wall space, and the LITA was harvested under direct visualization (Fig. 1A). For patients that were not able to tolerate single-lung ventilation, intermittent full-lung ventilation, decreased tidal volume, increased respiratory rates, and wet gauze placement to press the lung tissue were employed as effective alternative approaches. Chest wall retractors have both longer and shorter lift options. To achieve a maximal length, the retractor could be reversed to reach the inferior portion of the LITA. Systemic heparin (1.5 mg/kg) was applied, and ACT (Activated Clotting Time) was monitored and prolonged to a minimum of 300 s during anastomosis. Following pericardial incision and suspension, LITA-LAD anastomosis was performed with a tissue stabilizer (TS2000, Medtronic Inc., Minneapolis, MN, USA) and a ValveGate™ Hinged retractor (Fig. 1B). The right pericardium near the ascending aorta was suspended sufficiently using three to four stitches externalizing through the second intercostal space. The left pericardium was carefully retracted to avoid LITA strain. The the plane between the aorta and pulmonary artery was freed, and a gauge was placed at the right side of the aorta. Owing to patient-specific differences in angle or depth, a customized vascular clamp (Cardiomedical GmbH, Langenahgen, Germany) was placed on the ascending aorta (Fig. 1C) for proximal anastomosis using a long needle holder, ring tissue forceps, and a knot pusher (ValveGate™PRO) (Video). Distal anastomoses were then performed as in the OPCABG procedure. The exposure of the obtuse marginal branch (OM) is clearer under direct left intercostal visualization than during sternotomy as the target vessel is nearer to the incision, but in some cases, an Octopus stabilizer (29800 Medtronic Inc., Minneapolis, MN, USA) was used for the exposure of deeper sites such as the right coronary artery (RCA) main branch (Fig. 1D). TTFM (transit time flow measurement) was used as a routine approach to measuring immediate graft patency following vascular anastomosis, and was assessed with a QuickFit TTFM probe (Medistem VeriQ, Oslo, Norway). A single drainage tube was placed in the left thoracic cavity through the 6th–7th intercostal space before closure. The same dual antiplatelet regimen used for patients in the OPCABG group was applied to these patients.

### 6-Month coronary computed tomography angiography follow-up

Patient follow-up was conducted at 6 months post-surgery through clinical visits to outpatient departments and through WeChat interviews with patients and their relatives. Short-term graft patency was assessed via 6-month CT angiography (CTA) analysis, with the grades and patency of both LITA and vein grafts being defined using Fitzgibbon scores [15]. Unimpaired grafts were defined with a patency rating of ‘A’, while those with impaired runoff and with stenosis to less than 50% of the graft were defined with a patency rating of ‘B’, and a rating of ‘O’ was indicative of occlusion or suspicious underdevelopment.

### Statistical analysis

As the operative approach was a matter of subjective choice, group assignments were not random. Propensity score (PS) matching was performed in an effort to reduce the impact of group selection bias. To that end, epidemiological data, preoperative clinical characteristics, and angiographic lesions were used to match patients in the MICS CABG group to those in the OPCABG group at a 1:1 ratio via a nearest-neighbor matching algorithm, with a logistic regression estimation algorithm and a caliper width equal to 0.1 standard deviations (SDs) as the logit for PS matching. Matching was performed based on 17 covariates (Table 1), including age, gender, body mass index, left ventricular end-diastolic and systolic diameter, ejection fraction, LITA ultrasound results, CAG results of target vessels number and SYNTAX score, history of percutaneous coronary intervention (PCI), myocardial infarction, hypertension, diabetes, chronic lung disease, abnormal signs of cerebrovascular disease from head CT scans, and preoperative creatinine. Variables were considered to be well balanced at a standardized mean difference value of < 10% [16]. Conditional logistic regression analyses were performed to compare procedural characteristics and surgical outcomes between matched groups. PS matching was conducted using the psmatching 3.04 SPSS-R plugin 22.0 for R 2.15.X (MatchIt, Ritools, and cem) [17]. Continuous variables are given as means ± standard deviation, and categorical variables are given as numbers (percentages). Categorical variables were compared using chi-squared or Fisher’s exact tests. Quantitative variables were compared using the paired-samples t-tests. A P-value < 0.05 was considered statistically significant. SPSS v22 (SPSS, Inc., IBM, Chicago, IL) was used for all statistical analyses.

**Table 1:** Baseline characteristics before and after PS matching

Characteristics	Unadjusted			After PS matching		
	OPCABG (n = 371)	MICS (n = 211)	Standardized Difference	OPCABG (n = 172)	MICS (n = 172)	Standardized Difference
Age	61.6 ± 8.8	58.6 ± 9.3	33.10	60.2 ± 9.1	60.2 ± 8.9	0.00
Male	274 (74)	185 (88)	35.64	140 (81)	146 (85)	9.30
BMI	25.7 ± 3.4	25.4 ± 3.0	7.91	25.4 ± 3.0	25.6 ± 3.0	7.03
LVEDD	50.0 ± 6.5	48.8 ± 4.4	21.32	48.3 ± 6.0	49.1 ± 4.5	9.27
LVEDS	33.9 ± 6.9	32.1 ± 4.3	32.54	31.8 ± 5.2	32.5 ± 4.4	9.87
EF	57.8 ± 10.0	62.1 ± 6.0	52.70	62.0 ± 7.4	61.1 ± 5.7	9.90
LITA D	0.21 ± 0.04	0.21 ± 0.03	0.00	0.21 ± 0.03	0.21 ± 0.03	0.00
LITA V	74.6 ± 19.6	74.1 ± 19.1	2.79	75.1 ± 21.4	74.3 ± 19.9	5.70
Target vessels	3.2 ± 0.7	2.9 ± 0.8	41.81	3.1 ± 0.7	3.0 ± 0.8	9.83
SYNTAX score	45.6 ± 15.3	40.5 ± 15.9	32.51	42.6 ± 14.0	42.2 ± 15.8	2.41
History of PCI	55 (15)	39 (18)	9.84	27 (16)	30 (17)	4.68
History of MI	65 (18)	35 (17)	2.47	28 (16)	31 (18)	4.62
Hypertension	232 (63)	122 (58)	9.63	105 (61)	104 (61)	1.19
Diabetes	146 (39)	82 (39)	1.00	66 (38)	70 (41)	4.77
CLD	63 (17)	34 (16)	2.34	27 (16)	32 (19)	7.70
Cerebrovasc	58 (16)	33 (16)	0.03	27 (16)	26 (16)	1.63
Creatinine	80.1 ± 71.4	73.7 ± 18.2	12.25	74.1 ± 18.9	74.4 ± 19.5	1.56

*BMI* body mass index (kg/m.^2^), *LVEDD* left ventricular end diastolic diameter (cm), *LVEDS* left ventricular end systolic diameter (cm), *EF* ejection fraction (%), *LITA D* diameter of LITA (cm), *LITA V* velocity of LITA (cm/s), *Target vessels* target vessels number, *MI* myocadial infarction, *CLD* chronic lung disease history, *Cerebrovasc* cerebrovascular abnormal sign of head CT, *Creatinine* preoperative creatinine (umol/L)

## Results

From January 2017-January 2021, 582 patients with multi-vessel lesions were enrolled in the present study and underwent either MICS CABG or OPCABG treatment. There were significant imbalances in baseline demographic and clinical characteristics between these groups (Table 1), including age, gender difference, echocardiography index, target vessel number, and SYNTAX score (standardized difference > 10%). As such, the MICS CABG group was matched 1:1 with the OPCABG group via a PS matching approach based on 17 variables (MICS = 172; OPCABG = 172). In the matched groups, epidemiological data, preoperative clinical characteristics, angiographic lesions, and other covariates were well-balanced (standardized difference < 10%). MICS CABG group patients achieved mean graft numbers of 3.0 ± 0.8 covering all three main arterial territories, thereby achieving complete revascularization with a low need for circulatory assistance through femoral bypass or conversion to sternotomy (Table 2). Mean operative duration and duration of anesthetization were longer in the MICS CABG group relative to the OPCABG group, while ventilation and ICU (intensive care unit) duration were similar between these groups. Next day postoperative hemoglobin [(111.3 ± 16.3 g/L) vs. (107.1 ± 16.3 g/L), p = 0.044] was higher in the MICS CABG group. Three cases in the MICS CABG group were combined with other procedures. In one case, chest CT scans revealed an upper left lung mass, which was confirmed as a tuberculoma following concomitant resection, while the other two cases were thymoma. Two concomitant left lung mass resection procedures were performed in the OPCABG group.

**Table 2:** Surgical data of paired groups

Characteristics	OPCABG (n = 172)	MICS (n = 172)	P value
Total operation time (min)	215.9 ± 48.8	308.6 ± 77.9	< 0.001
LITA use	161 (94)	164 (95)	0.479
LAD	170 (99)	166 (97)	0.283
Diag	51(30)	80 (47)	0.001
Ramus	15 (8.7)	13 (7.6)	0.693
OM	131 (76)	122 (71)	0.271
PLA	15 (89)	21 (12)	0.291
PDA	115 (67)	85 (49)	0.001
RCA	25 (14.5)	12 (7.0)	0.024
Endarterectomy	7 (4.1)	3 (1.7)	0.336
Conversion to CPB	6 (3.4)	3 (1.7)	0.494
Conversion to sternotomy	–	1 (0.6)	–
Intraoperative bleeding (ml)	743.8 ± 428.3	720.83 ± 325.1	0.677
Postoperative drainage (ml)	453.7 ± 253.2	407.5 ± 267.8	0.101
Next day postoperative hemoglobin (g/L)	107.1 ± 16.3	111.3 ± 16.3	0.044
Combined procedure	2 (1.2)	3 (1.7)	0.657
Ventilation duration (hrs)	18.7 ± 12.3	17.7 ± 13.4	0.643
ICU duration (hrs)	22.8 ± 15.4	21.54 ± 14.3	0.569

Data are depicted as number and percentage or mean ± SD

*LAD* left anterior descending artery, *Diag* diagonal branch, *OM* obtuse marginal branch, *PLA* posterior branch of left ventricle, *PDA* posterior descending artery, *RCA* right coronary artery, *CPB* cardiopulmonary bypass

Next, 30-day postoperative outcomes in the matched patient groups were compared (Table 3). No differences in mortality, myocardial infarction, or stroke rates were observed between these groups. One death occurred in the MICS CABG group due to ventricular tachycardia after coronary endarterectomy. There were no significant differences in rates of reoperation or atrial fibrillation. Intra-aortic balloon pumps (IABPs) were used in 3 and 2 cases in the MICS CABG and OPCABG groups. Continuous renal replacement therapy (CRRT) was used in the OPCABG group, extracorporeal membrane oxygenation (ECMO) was used in both groups. Average postoperative hospital duration was lower in the MICS CABG group [(6.2 ± 1.4) vs. (6.9 ± 2.6) days, p = 0.023], while Barthel Index for ADL scores prior to discharge were higher in the MICS CABG group (P < 0.05).

**Table 3:** Postoperative outcomes

Characteristics	OPCABG (n = 172)	MICS (n = 172)	OR (95% CI)	P value
30-day mortality	1 (0.6)	1 (0.6)	1.00 (0.06–15.99)	> 0.999
MI	2 (1.2)	3 (1.7)	1.50 (0.25–8.98)	0.657
Stroke	1 (0.6)	1 (0.6)	1.00 (0.06–15.99)	> 0.999
Reoperation	4 (2.3)	2 (1.2)	0.50 (0.09–1.73)	0.423
Atrial fibrillation	34 (19.8)	35 (120.4)	1.03 (0.64–1.65)	0.904
Transfusion	6 (3.5)	2 (1.2)	0.33 (0.07–1.65)	0.178
CRRT	1 (0.6)	0 (0)	–	–
IABP	2 (1.2)	3 (1.8)	1.50 (0.25–8.98)	0.657
ECMO	1 (0.6)	1 (0.6)	1.00 (0.06–15.99)	> 0.999
Postoperative hos. stays	6.9 ± 2.6	6.2 ± 1.4	–	0.023
Barthel Index for Activities of Daily Living before discharging	41.7 ± 21.6	51.8 ± 21.2	–	< 0.001

Data are depicted as number and percentage or mean ± SD

*OR* odds ratio, *CI* confidence interval, *MI* myocardial infarction, *CRRT* continuous renal replacement therapy, *IABP* intra-aortic balloon pump, *ECMO* extracorporeal membrane oxygenation, *Postoperative hos. Stay* postoperative hospital stays (days)

At the 6-month follow-up time point, of the 344 patients in the matched cohort, 60 failed to undergo CTA (17.4%). The remaining 861 grafts in these patients were available for 6-month short-term follow-up evaluation. The overall 6-month graft patency rates in the MICS CABG and OBCABG groups were 92.0% and 92.7% (p = 0.780), respectively, with LITA graft patency rates of 95.0% and 96.1% (p = 0.646), respectively, and vein graft patency rates of 90.6% and 91.2% (p = 0.785), respectively (Table 4). No difference in short-term graft patency was observed when comparing these groups.

**Table 4:** 6 month follow-up of CTA

Characteristics	OPCABG (n = 172)	MICS (n = 172)	P value
Patients with follow-up CTA	138	146	–
Total grafts number	425	436	–
LITA grafts	129	139	–
SVG grafts	296	297	–
Fitzgibbon grade A	376	387	–
Fitzgibbon grade B	18	14	–
Fitzgibbon grade O	31	34	–
Total graft patency (%)	92.7	92.0	0.780
LITA patency (%)	96.1	95.0	0.646
SVG patency (%)	91.2	90.6	0.785

Data are depicted as number and percentage

*CTA* computed tomography angiography, *SVG* saphenous vein graft

## Discussion

Over the past three decades, there has been a growing demand for the development of efficient, safe, and effective minimally invasive approaches to surgical CAD treatment. For CAD patients with multi-vessel disease, MICS CABG procedures have been performed for almost two decades and can achieve full myocardial revascularization via a less invasive single small intercostal incision approach, yielding faster recovery relative to conventional procedures [12, 13, 18–20]. However, many surgeons have expressed concern regarding the safety of this procedure owing to unfamiliarity with this technique and the need for more advanced surgical techniques and specialized instrumentation when conducting MICS CABG. The present PS-matched study based on SYNTAX score was conducted to assess perioperative outcomes and short-term graft patency in patients that had undergone multi-vessel conventional OPCABG or MICS CABG treatment through the left intercostal space.

In this study, we found that MICS CABG was able to cover all arterial territories and to achieve complete revascularization, with only one case of conversion to sternotomy and three cases in which femoral circulatory assistance was required. The 30-day mortality, myocardial infarction, and stroke rates were similar for patients in the OPCABG and MICS CABG groups. One patient experienced myocardial infarction due to intraoperative ventricular tachycardia after endarterectomy. Despite cardiopulmanry bypass (CPB) and ECMO, this patient died as a consequence of sepsis and multiple organ dysfunction syndrome. MICS CABG offered advantages of shorter postoperative hospitalization and an earlier return of physical function. Overall half-year graft patency rates did not differ significantly between the MICS CABG and OPCABG groups (92.0% vs. 92.7%, p = 0.780). Proper patient selection and quality control strategies are an important part of surgical planning. Approaches adopted to improve MICS CABG safety include the following: (1) LITA-LAD anastomosis is routinely prioritized in order to achieve early LAD revascularization as a means of guaranteeing the safety of the proximal anastomosis procedure. This sequence does not increase the difficulty of the proximal anastomosis of the ascending aorta, and further improves the safety of subsequent operations. (2) During the off-pump procedure, a coronary intraluminal shunt is routinely used to minimize the risk of myocardial ischemia–reperfusion injury [21, 22]. Such coronary intraluminal shunt use can safely ensure smooth hemodynamic flow. TTFM was used as a reliable measure to define immediate graft patency following each anastomosis after protamine neutralization. This is an important means of predicting acute myocardial infarction in the perioperative period, and instant patency during bypass surgery is the most critical predictor of short- and long-term clinical outcomes. (3) Anastomosis of the great saphenous vein and the ascending aorta is the most difficult aspect of this procedure. Approaches such as total arterialization or sequential venous bridge anastomosis can be used to avoid multiple proximal anastomoses to the ascending aorta. (4) During the anastomosis of the great saphenous vein and the ascending aorta, efforts are made to avoid interfering with pulmonary artery blood flow by fully freeing the aorticopulmonary septum, packing gauze on the right side of the ascending aorta, and applying a customized vascular clamp. Approaches to obtaining good exposure of the ascending aorta can avoid overload of the right ventricular system or decreases in pulmonary blood flow [23, 24].

With respect to patient selection, both preoperative chest CT scans and peripheral vascular ultrasonography are essential. Chest CT scans can enable the direct evaluation of aortic calcification while simultaneously offering insight into potential lung conditions such as fibrosis, emphysema, infection, or history of prior surgery. In one case in this study, chest CT scans revealed the presence of an upper left lung mass (2 × 3 cm) that was later pathologically confirmed to be a tuberculoma following its resection. No instances of stroke occurred among patients in the MICS CABG group, potentially due to the exclusion of patients with aortic calcification and severe stenosis of the carotid and vertebral arteries, and to the relatively high degree of blood pressure control implemented during proximal anastomosis.

## Conclusions

With proper patient selection, MICS CABG can achieve perioperative results and short-term graft patency comparable to those associated with conventional OBCABG. While this minimally invasive procedure is associated with a longer average operative duration, it also offers advantages including shorter postoperative hospitalization and an earlier return of physical function. Higher levels of postoperative hemoglobin and bone no-touch technique were also associated with faster recovery. As such, MICS CABG represents a promising and widely applicable approach to treating patients with multi-vessel lesions that is likely to gain increasing adoption by cardiac surgeons. We believe that MICS CABG is a less traumatic alternative to complete revascularization.

### Limitations

There are certain limitations to this study. This was a retrospective analysis, and patient group assignments were not random and were instead made based on the clinical decisions of the operating surgeon. We adjusted for bias using a case–control design, matching MICS CABG and OPCABG patients at a 1:1 ratio based on preoperative variables and graft number. However, unmeasured variables may have influenced comparisons between groups. Moreover, only limited follow-up outcomes were assessed in this study, and additional mid-term and long-term follow-up research is necessary. Additionally, this was a single-center study, and additional multi-center evidence is necessary to validate these findings.

## Data Availability

The datasets used or analysed during the current study are available from the corresponding author on reasonable request.

## Abbreviations

MICS CABG: Minimally invasive cardiac surgery coronary artery bypass grafting
OPCABG: Off-pump coronary artery bypass grafting
MIDCAB: Minimal invasive direct coronary artery bypass grafting
LITA: Left internal thoracic artery
PSM: Propensity score-matched
ADL: Activities of daily living
TTFM: Transit time flow measurement
PS: Propensity score
IABP: Intra-aortic balloon pump
CRRT: Continuous renal replacement therapy
ECMO: Extracorporeal membrane oxygenation
